# Urinary π-glutathione S-transferase Predicts Advanced Acute Kidney Injury Following Cardiovascular Surgery

**DOI:** 10.1038/srep26335

**Published:** 2016-08-16

**Authors:** Kai-Hsiang Shu, Chih-Hsien Wang, Che-Hsiung Wu, Tao-Min Huang, Pei-Chen Wu, Chien-Heng Lai, Li-Jung Tseng, Pi-Ru Tsai, Rory Connolly, Vin-Cent Wu

**Affiliations:** 1 Division of Nephrology, Department of Internal Medicine, National Taiwan University Hospital, Taipei, Taiwan; 2 NSARF group (National Taiwan University Hospital Study Group of ARF), Taipei, Taiwan; 3 Division of Nephrology, Department of Internal Medicine, Far Eastern Memorial Hospital, New Taipei City, Taiwan; 4 Department of Surgery, National Taiwan University Hospital, Taipei, Taiwan; 5 Division of Nephrology, Department of Internal Medicine, Taipei Tzu Chi Hospital, New Taipei City, Taiwan; 6 Division of Nephrology, Department of Internal Medicine, National Taiwan University Hospital Yun-Lin Branch, Douliou, Taiwan; 7 Division of Nephrology, Department of Internal Medicine, Mackay Memorial Hospital, Taipei, Taiwan; 8 EKF Diagnostics, Cardiff, Wales, UK

## Abstract

Urinary biomarkers augment the diagnosis of acute kidney injury (AKI), with AKI after cardiovascular surgeries being a prototype of prognosis scenario. Glutathione S-transferases (GST) were evaluated as biomarkers of AKI. Urine samples were collected in 141 cardiovascular surgical patients and analyzed for urinary alpha-(α-) and pi-(π-) GSTs. The outcomes of advanced AKI (KDIGO stage 2, 3) and all-cause in-patient mortality, as composite outcome, were recorded. Areas under the receiver operator characteristic (ROC) curves and multivariate generalized additive model (GAM) were applied to predict outcomes. Thirty-eight (26.9%) patients had AKI, while 12 (8.5%) were with advanced AKI. Urinary π-GST differentiated patients with/without advanced AKI or composite outcome after surgery (p < 0.05 by generalized estimating equation). Urinary π-GST predicted advanced AKI at 3 hrs post-surgery (p = 0.033) and composite outcome (p = 0.009), while the corresponding ROC curve had AUC of 0.784 and 0.783. Using GAM, the cutoff value of 14.7 μg/L for π-GST showed the best performance to predict composite outcome. The addition of π-GST to the SOFA score improved risk stratification (total net reclassification index = 0.47). Thus, urinary π-GST levels predict advanced AKI or hospital mortality after cardiovascular surgery and improve in SOFA outcome assessment specific to AKI.

In a physician’s daily clinical practice, the evaluation of kidney function is certainly part of the comprehensive care for patients, with collection of numerous data points for association of acute kidney injury (AKI) and adverse clinical outcome^1^. Creatinine is certainly a useful tool for evaluation of kidney excretory function. However, it tells little about whether structural damage coexists or precedes the functional change. Urinary biomarkers have been proposed to predict and/or augment the diagnosis of AKI in order to overcome the limitations inherent to creatinine and urine output criteria, i.e., inadequate sensitivity and delayed response. A variety of serum and urinary biomarkers have been under extensive investigation and validation^2^ since the pioneer study series of neutrophil gelatinase-associated lipocalin (NGAL) more than a decade ago^3^,^4^. Biomarkers of AKI could be used to evaluate the severity of AKI at an earlier time point in the course of the disease, defining those with high risk of progression to more advanced kidney injury. Moreover, there is urgent need to use novel biomarkers as a guide for clinical decision-making, and augmenting selection of effective management strategies for advanced AKI, e.g. dialysis. The list of candidate biomarkers is ever-growing^5^, including but not limited to neutrophil gelatinase-associated lipocalin (NGAL), liver fatty acid-binding protein (L-FABP), kidney injury molecule 1 (KIM1), interleukin-18 (IL-18), cystatin C, and the combination of tissue inhibitor of metalloproteinase-2 (TIMP-2) and insulin-like growth factor binding protein 7 (IGFBP7), either serum or urine concentration. However, the predictive performance is variable, while the heterogeneous end-point definitions complicate the comparison. In order to determine the diagnostic yield of various biomarkers^6^, evaluating AKI after cardiovascular surgery became the prototype scenario for both development and validation purposes^7^. Glutathione S-transferase (GST), a constitutive cytoplasmatic enzyme, enriches in the renal tubular epithelial cells and is detectable in the urine when the cell integrity of renal tubules is damaged. As a scavenger of free radicals, GST may help tubular epithelial cells withstand stressful conditions. GST was originally reported to be a tubular injury marker elevated under renal insults^8^,^9^. Further immunohistochemistry examinations demonstrated the localization of α- and π-GST in the proximal and distal renal tubules, respectively^10^. In the era of AKI urinary biomarkers, GST gained its position in several well-known kidney injury models^11^,^12^,^13^,^14^,^15^,^16^, although the discriminating power, mostly expressed as area under the receiver operating characteristic (ROC) curve^17^ was variable, while the optimal timeframe of marker measurement was not well established. The performance and best cut-off value of both α- and π-GST are thus evaluated prospectively in this study, in order to elucidate their respective performance as critical AKI biomarkers, in terms of dialysis or mortality.

## Materials and Methods

### Study Population

This study was conducted by the biomarker investigators from NSARF (the National Taiwan University Study Group on Acute Renal Failure)^18^,^19^,^20^,^21^,^22^, a multi-center, prospectively constructed database of AKI. From 2009 to 2010, patients having cardiovascular surgery, including coronary bypass and valvular operations, were enrolled prospectively from tertiary centers in northern Taiwan and an affiliated local hospital in central Taiwan. Patients with the following conditions were excluded: those who had undergone renal replacement therapy, those who were diagnosed as AKI by KDIGO definitions^23^ during index hospitalization before operation, those who were younger than 18 years of age, had a history of renal transplantation or nephrectomy, and estimated glomerular filtration rate <30 ml/1.73 m^2^ at the time of ICU enrollment. Availability of a baseline serum creatinine level 7 to 180 days prior to the index hospital admission was mandatory. The institutional research ethic review board of National Taiwan University Hospital approved the data collection of this study (201105040RC). This research was carried out in accordance with the approved guidelines. Written informed consent was obtained from subjects.

### Clinical Data Collection

Relative clinical information was obtained, including demographic data, intervention procedures, and comorbidity status. Urine output and creatinine levels were recorded at each time point after operation as detailed in the study protocol. Post-operative inotropic agent use was quantified as per inotropic equivalents^19^,^21^,^24^. Operation related parameters included operation method, clamping time of aorta, and cardiopulmonary bypass time. Lengths of total admission and ICU admission were also recorded. Disease severity was evaluated as Sequential Organ Failure Assessment (SOFA) score^25^.

### Sample Collection

Urine samples were obtained in the ICU at 3, 6, 9, 12, and 24 hours post-surgery. Other laboratory examinations were performed as indicated clinically. Urine specimens were collected by standardized procedure, centrifuged within 1 hour and the sediments discarded. The urine samples, collected in separate polypropylene tubes containing sodium azide, were stored at −80 °C until required. Each specimen was centrifuged at 800 g at 4 °C for 5 minutes, and the supernatant was collected for an ELISA assay.

### Biomarker Measurements

The urinary α-GST and π-GST levels were determined by EKF Diagnostics Human Alpha and Pi GST EIA Test Kits (product BIO91 and BIO85, respectively). The results were expressed in μg/mL. The inter- and intra-assay coefficient of variation for α-GST was 6.3% and 2.7%, respectively, and for π-GST, 8.6% and 3.1%, respectively. The lower limit of detection for α-GST and for π-GST was 0.3 μg/mL. Assays were completed as described by the manufacturer’s protocol, and each measurement was performed in duplicate. Urine creatinine levels were measured using the Jaffe assay, with standardization to isotope dilution mass spectrometry (IDMS)-traceable reference.

### Outcome Definitions

Clinical endpoint was defined as the occurrence of advanced AKI (stage 2 or 3, specified as the Kidney Disease: Improving Global Outcomes KDIGO classification^23^). Both urine and creatinine criteria applied. The baseline creatinine measurement was defined as pre-admission baseline creatinine, where available, or the first creatinine measurement in the emergency department. The composite outcome of developing stage 2 or 3 AKI and in-hospital mortality was also analyzed. Patients were followed until the time of death or hospital discharge, whichever earlier.

### Statistics Analyses

All analyses were performed with SPSS software, version 19 (IBM, Armonk, NY) and R software^26^. To compare continuous variables, a two-tailed unpaired *t* test was utilized; for categorical variables, the χ^2^ or Fisher’s exact test were applied. To measure performance of urinary GST, a receiver-operating characteristic (ROC) curve was generated, with further calculation of the area under the curve (AUC). To assess the performance of the biomarker over the clinical model, we calculated the improvement in Harrell C statistic, accounting for censoring in survival biomarker concentrations, and transformed using natural logarithm if necessary before adding individually to the clinical model.

To examine the effect of AKI on various time-dependent GST variables over the sequential changes of time, marginal linear regression models were fitted to these repeatedly measured responses using the generalized estimating equation (GEE) method^24^,^27^. In fitting logistic regression model using the GEE method, the first-order autocorrelation was chosen as the working correlation structure, and then the robust standard error was chosen to compute the p value. Standardized regression coefficients and their 95% confidence intervals (CI) were calculated. All the univariate significant and non-significant relevant covariates (listed in Table 1) and some of their interactions were put on the variable list to be selected. The significance levels for entry (SLE) and for stay (SLS) were set to 0.15 for being conservative. Then, with the aid of substantive knowledge, the best candidate final logistic regression model was identified manually by dropping the covariates with p value >0.05 one at a time until all regression coefficients were significantly different from 0.

In order to display the implications of GST for individual patients, a generalized additive model (GAM) (with spline) incorporating the subject-specific (longitudinal) random effects was plotted and adjusted for other clinical parameters to predict the outcomes^28^,^29^. Simple and multiple generalized additive models (GAMs) were fitted to detect nonlinear effects of continuous covariates and identify appropriate cut-off point(s) for discretizing continuous covariates, if necessary, during the stepwise variable selection procedure. We defined the optimal cut-off value as log odd equals to zero^30^. The vgam function (with the default values of smoothing parameters) of the VGAM package^31^,^32^,^33^ was used to fit GAMs for the binary responses in R.

Moreover, the ability of π-GST to more accurately stratify individuals into higher or lower risk categories (reclassification) was investigated by net reclassification improvement (NRI) and integrated discrimination improvement (IDI). An increase in NRI was calculated in a model with sequential organ failure assessment score (SOFA) and π-GST, compared with SOFA risk factors alone. We reclassified the patients who died from all-causes or who developed advanced AKI using 0–8%, 8–30%, and >30% for the risk categories. A p-value less than 0.05 was considered significant.

## Results

### Clinical Characteristics

A total of 141 patients receiving cardiovascular surgery were eligible for the study, with subsequent time-varying sample collection. Thirty-eight patients suffered from AKI among 141 subjects (26.9%), with 26 (18.4%) having stage 1 AKI. Seven patients (5.0%) developed stage 2 AKI, and 5 patients (3.5%) progressed to stage 3. The clinical characteristics of patients with and without advanced AKI (defined as KDIGO stage 2 or 3) are described in Table 1. The underlying comorbidities were comparable in both groups. Advanced AKI patients had lower hemoglobin and albumin levels. Mean serum creatinine level and estimated glomerular filtration rate were not different between patients with or without advanced AKI. The in-hospital mortality rate in this cohort was 5.7%. The advanced AKI group was highlighted by higher clinical severity measured by the 24-hour SOFA score, where this critical condition translated into a longer ICU stay and higher in-hospital mortality rate. Those without any AKI had a mean π-GST level of 35.9 μg/L at 3 hours after operation, while the mean in those with any AKI was 131.1 μg/L (p = 0.084 by two-tailed *t* test).

### Model Prediction of Stage 2 or 3 AKI

**Figure 1:**
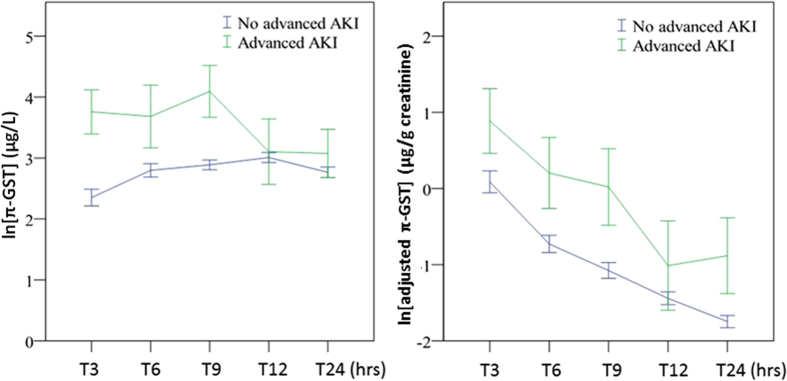
(**A**) Urinary π-GST levels for patients with/without advanced AKI (KDIGO stage 2 or 3) (over time, p < 0.001). (**B**) Adjusted urinary π-GST levels for patients with/without advanced AKI (over time, p = 0.005). All expressed as mean ± standard error of mean.

**Figure 2:**
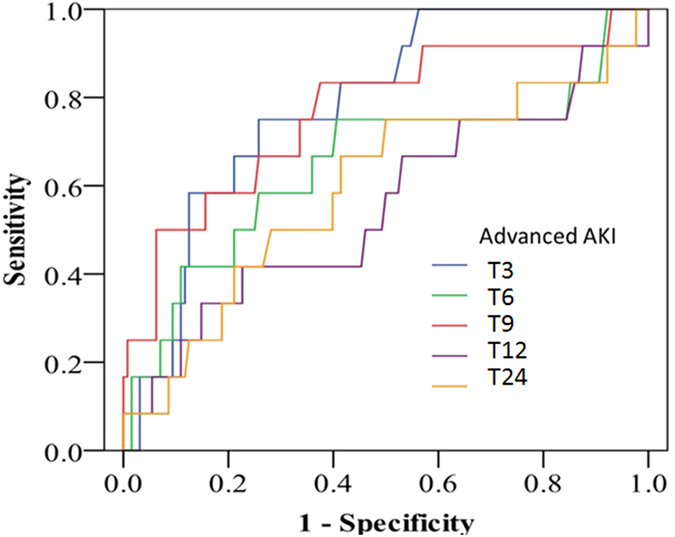
ROC curves for urinary π-GST levels predicting advanced AKI. Curves for different time points labeled as 3 hours [T3], 6 hours [T6], 9 hours [T9], 12 hours [T12], and 24 hours post-surgery [T24].

The respective π-GST levels are shown in Fig. 1, grouped as with/without advanced AKI (KDIGO stage 2 or 3), at various post-operative timing points and with/without normalization to urinary creatinine. There was a trend toward π-GST elevation post operation in the advanced AKI group (by GEE, p < 0.001) but the difference disappeared if creatinine-adjusted π-GST was analyzed. Table 2 summarizes the AUCs for ROC curves generated for the diagnosis of advanced AKI by π-GST. The AUCs for π-GST at 3 hours and 9 hours post surgery were 0.784 (95% CI 0.673 to 0.895, p < 0.005) and 0.767 (95% CI 0.610 to 0.924, p < 0.005), respectively. At 3 hours, patients without advanced AKI had a mean π-GST level of 59.1 μg/L, while the mean in those with advanced AKI was 88.8 μg/L (p = 0.467 by two-tailed unpaired *t* test). Figure 2 illustrates the corresponding ROC curves for π-GST.

Compared to the positive result of π-GST, α-GST levels failed to show diagnostic accuracy by ROC curve or statistical significance between advanced AKI and non-advanced AKI groups by GEE model.

**Figure 3:**
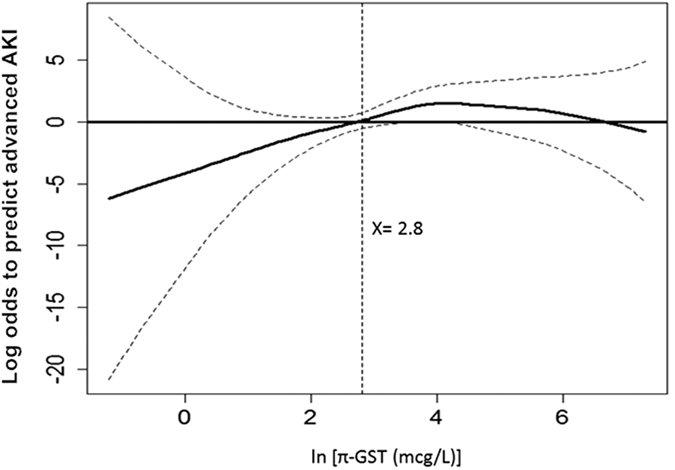
GAM plot for the probability of advanced AKI for urinary π-GST levels at T3 (3 hours post surgery). The model incorporates the subject-specific (longitudinal) random effects, expressed as the logarithm of the odd (logit). The probability of outcome events was constructed with π-GST level and was centered to have an average of zero over the range of the data as constructed with the GAM. Log [π-GST (μg/L)] = 2.8 was an independent factor predicting postoperative stage 2 or 3 AKI.

In the multivariable risk model, the independent risk factors to stage 2 or 3 AKI were urinary π-GST level at 3 hrs post surgery, inotropic equivalents, hemoglobin, and BMI (Table 3). The generalized additive model (GAM) plot was generated thereafter and demonstrated the positive correlation between increased urinary π-GST levels at 3 hrs post surgery and the risk of developing advanced AKI (Fig. 3). After adjusting the nonlinear effects of the variables, π-GST = 16.5 μg/L (ln [π-GST (μg/L)] = 2.8) independently predicted postoperative stage 2 or 3 AKI. The corresponding sensitivity and specificity were 75.0% and 68.2%, with positive and negative predictive values (PPV and NPV) being 18.0% and 96.7%, respectively.

In the present study, we also examined the performance of adjusted GST concentration, i.e. urinary GST concentration divided by urinary creatinine concentration. Nonetheless, adjustment by urinary creatinine failed to improve the diagnostic accuracy, although with a borderline significance for prediction (for urinary π-GST divided by urinary creatinine [adjusted π-GST] at 3 hrs post surgery, AUC of ROC curve 0.675, 95% CI 0.546 to 0.804, p < 0.05).

### Model Prediction of Composite Outcome of Stage 2 or 3 AKI or In-hospital Mortality

**Figure 4:**
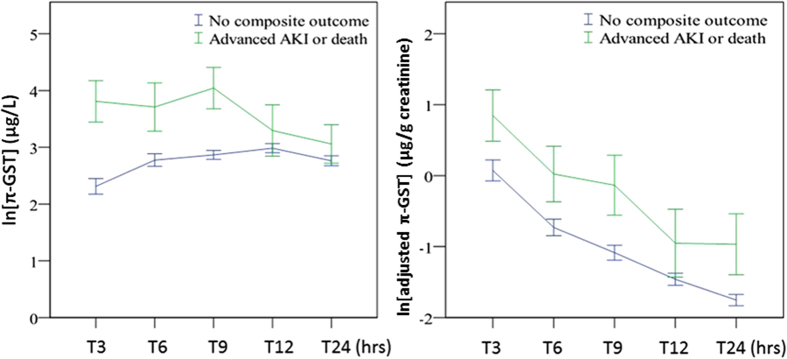
(**A**) Urinary π-GST for patients with/without the composite outcome of advanced AKI or in-hospital mortality (over time, p < 0.001). (**B**) Adjusted urinary π-GST levels for patients with/without the composite outcome (over time, p = 0.017). All expressed as mean ± standard error of mean.

**Figure 5:**
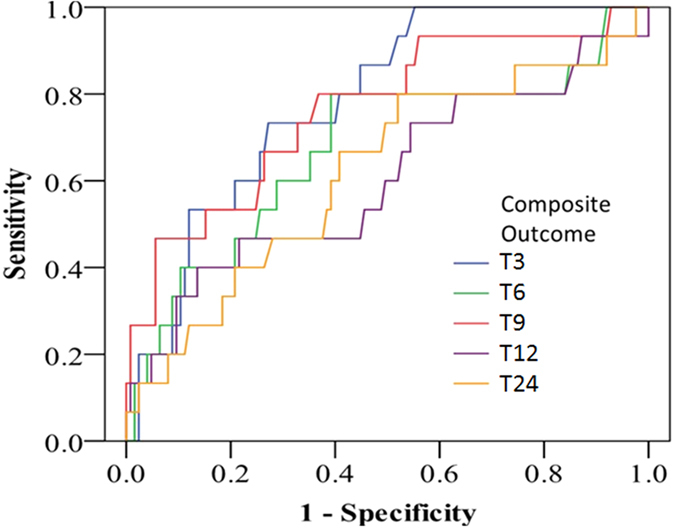
ROC curves for urinary π-GST levels predicting the composite outcome of advanced AKI or in-hospital mortality. Curves for various time points labeled as 3 hours [T3], 6 hours [T6], 9 hours [T9], 12 hours [T12], and 24 hours post-surgery [T24].

Figure 4 depicts the urinary π-GST levels for those with/without composite outcome, i.e., advanced AKI of KDIGO stage 2 or 3 or in-hospital mortality. The π-GST levels differ significantly in the two aforementioned groups by the analysis of GEE (over time, p < 0.001). The AUCs (Table 2) of ROC curves (Fig. 5) were remarkable for the significant predictive power of π-GST levels at various time points: AUC 0.783 at 3 hrs post surgery (95% CI 0.682 to 0.884, p < 0.005), AUC 0.675 for data at 6 hrs post surgery (95% CI 0.512 to 0.837, p < 0.05), and AUC 0.763 at 9 hrs post surgery (95% CI 0.626 to 0.900, p < 0.005). At 3 hours, patients without composite outcome had a mean π-GST level of 54.3 μg/L, while the mean in those with composite was 123 μg/L (p = 0.231 by two-tailed unpaired *t* test).For cases of advanced AKI, the adjustment of urinary creatinine level failed to increase the diagnostic accuracy of urinary π-GST. Neither α-GST nor creatinine-adjusted α-GST levels were able to differentiate those with/without composite events.

**Figure 6:**
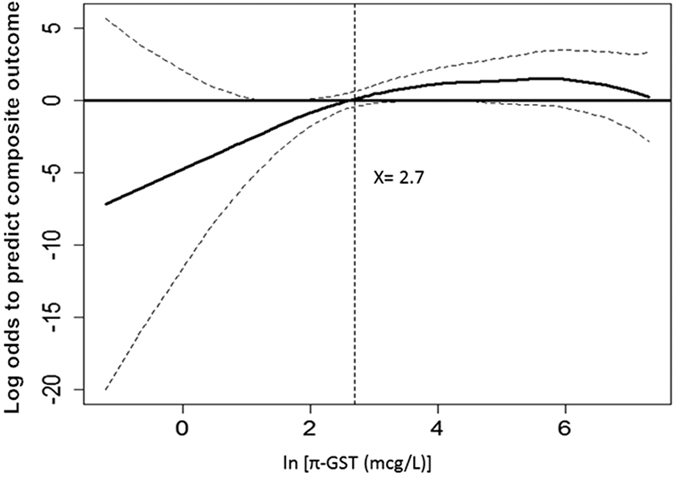
GAM plot for the probability of composite outcome (advanced AKI or in-hospital mortality) for urinary π-GST levels at T3 (3 hours post surgery). The model incorporates the subject-specific (longitudinal) random effects, expressed as the logarithm of the odd (logit). The probability of outcome events was constructed with π-GST level and was centered to have an average of zero over the range of the data as constructed with the GAM. Log [π-GST (μg/L)] = 2.7 was an independent factor predicting postoperative stage 2 or 3 AKI or in hospital mortality.

Table 3 demonstrates the multivariable model generated for predicting the composite outcome of advanced AKI or death by π-GST. Independent variables were applied to generate the GAM graph (Fig. 6), in which π-GST levels at 3 hrs post surgery were plotted against the log of the odd of the probability for composite outcome. The cutoff value in the GAM model, i.e., π-GST = 14.7 μg/L, (ln [π-GST (μg/L)] = 2.7), translated into a sensitivity of 73.3% and a specificity of 66.7%, while PPV and NPV in our studied population were 20.8% and 95.5%, respectively.

In relation to composite outcome, we examined reclassification for detection of advanced AKI or death. The addition of π-GST to the SOFA score led to a significant increase in risk stratification (total NRI = 0.47; 95% CI, 0.22 to 0.73; P < 0.001). The majority of this effect came from those with death or dialysis events (NRI event = 0.29; 95% CI, 0.05 to 0.52; P < 0.001), whereas the NRI non-event was 0.19 (95% CI, 0.10 to 0.28; P = 0.38). Similarly, the total integrated discrimination improvement (IDI) was significant at 0.16 (95% CI, 0.07 to 0.24; P < 0.001).

## Discussion

In the present study, we evaluated the proximal tubule enzyme α-GST and distal tubule enzyme π-GST as urinary biomarkers for AKI prediction. After cardiovascular surgery, the π-GST level at 3 hrs post surgery showed a higher discrimination power than α-GST level to predict advanced AKI and/or in-hospital mortality. We further identified the optimal cutoff value for π-GST to predict advanced AKI and composite outcome according to GAM plot, where the log odds for the outcome equals zero. The addition of π-GST to the SOFA score could lead to a significant improvement in outcome stratification.

Considering the analysis for stage 2 or 3 AKI, significant AUCs were obtained by using π-GST level at 3 hrs post surgery. This more severe kidney injury may reflect early extensive tubular dysfunction in the distal renal tubule or damage at AKI occurrence, further amplifying the discrepancy to milder forms of AKI or those without clinical kidney injury. This supports the view that these markers are for early diagnosis of AKI and for “early” diagnosis only. Susantitaphong *et al*. reported GST could predict advanced AKI at 2 hrs following cardiopulmonary bypass (AUC = 0.76)^16^. In Koyner’s report^34^, π-GST could predict the progression to stage 3 AKI at 6 hr post surgery in 123 adults undergoing cardiac surgery (AUC = 0.78). We further showed π-GST predicts stage 2 and 3 AKI at 3 hrs post surgery (AUC = 0.78). Collectively, these results supported the finding that GST is very sensitive to renal hypoperfusion, with early release even after mild injury^35^. Due to its rapid increase in urinary concentration, its applicability might be more appropriate in a setting that monitors the renal toxic effects of drugs and contrast agents in the kidneys^34^,^36^. This could be valuable for clinical use to identify the timing regarding the dialysis initiation and, identifying those patients with AKI who have severe tubular injury and will require further renal support pending recovery, since the culprit cannot usually be known for sure in clinical practice. This notion is also supported by the ability of π-GST to predict dialysis requirement in patients with baseline eGFR <60 mL/min/1.73 m^2^ (AUC 0.61) and oliguria (AUC 0.72)^15^. The serial GST level change may be a trajectory of renal function change.

The better predictive value of π-GST compared to α-GST for established AKI in this study is in accordance with other work^15^,^16^. π-GST showed better performance than α-GST regarding advanced AKI and in hospital mortality after cardiovascular surgery. α-GST was frequently elevated even though the subject did not meet the diagnostic criteria of AKI. Whether this reflects false-positive results or a signal to subclinical AKI is not clear. Similar discrepancy was noted in a sepsis-induced AKI study^12^. Renal GST was upregulated transcriptionally in nephrotoxin-induced cell model^37^ and AKI rat model^38^, demonstrating not only an injury model but also with possible mechanistic relevance^39^. Whether the distal marker of π-GST outperformed the proximal marker of α-GST in representing the underlying mechanism of AKI remained to be elucidated, but it may reflect the nature of different etiologies of severe kidney insults.

In real world practice, we lack markers to determine which patients will develop advanced AKI and disease progression to severe outcomes. Our results indicate that addition of π-GST to the SOFA score could improve the accuracy to predict progression to severe outcome, such as death or advanced AKI. These results may be useful in designing therapeutic trials for AKI by targeting patients most likely to benefit.

In our data, adjustment to urinary creatinine concentration only further attenuated the diagnostic power. The ratio to the urine creatinine level reflects the hope to extrapolate the value to 24-hour excretion amount, requiring the creatinine level to be in a steady state. Contradictory to this, the serum creatinine is not under steady state during AKI periods^34^. Moreover, due to the significant augmentation of tubular creatinine secretion under the setting of decreased GFR, the presence of tubular damage further impaired creatinine secretion. Thus, the role of creatinine adjustment for urinary biomarkers is uncertain^40^. While it may occasionally enhance the diagnostic efficiency for various biomarkers in rats, π-GST was characteristic of high sensitivity in our study and holds particular promise to facilitate improved clinical decision in patients at risk of developing advanced AKI.

The strengths of this study include multi-center design and homogenous (post-cardiovascular surgery) patient population. By enrolling patients with known baseline creatinine levels, we also reduced misclassification of early AKI status. This finding further substantiates a biological link between GST and clinical disease. Improving risk prediction may facilitate participation in AKI trials, enhance patient care in the setting of critical illness, and guide patient decision making and counseling with regard to treatment plans. However, the limitation of the current study is that we did not design to compare or combine these biomarkers with other novel AKI biomarkers. The existing data serves as a developmental set for the diagnostic test however, external validation in other populations should be considered. In the era of multiple biomarker development, panels of biomarkers, including GST, should be validated for additional value in combining more than one biomarker. This may help us to find better roles for GSTs and other biomarkers^41^. Finally, although the performance of biomarkers under the setting of cardiovascular surgery is fairly well defined, extrapolating to other clinical scenarios may not be as successful^12^. The generalizability to medical rather than surgical patients remains an ongoing issue.

## Conclusion

In summary, our data demonstrated that π-GST may be a valuable tool for early diagnosis of advanced AKI and prediction of prognosis after cardiovascular surgery, whereas α-GST did not. π-GST could improve the predictive ability of SOFA for composite outcome. Further external validation in a larger population is necessary to confirm this finding.

## Additional Information

**How to cite this article**: Shu, K.-H. *et al*. Urinary π-glutathione S-transferase Predicts Advanced Acute Kidney Injury Following Cardiovascular Surgery. *Sci. Rep.* **6**, 26335; doi: 10.1038/srep26335 (2016).

## Figures and Tables

**Table 1:** Clinical characteristics.

	All (n = 141)	No AKI or stage 1 AKI (n = 129)	Stage 2 or 3 AKI (n = 12)	p value
Patient characteristics				
Age	62.4 ± 13.6	62.3 ± 13.7	63.4 ± 12.4	0.779
Gender (male)	98 (69.5%)	93 (72.1%)	5 (41.7%)	0.045
BMI	24.7 ± 3.6	24.7 ± 3.4	24.6 ± 5.3	0.894
Comorbidities				
Hypertension	72 (51.1%)	67 (51.9%)	5 (41.7%)	0.705
Diabetes mellitus	34 (24.1%)	33 (25.6%)	1 (8.3%)	0.293
COPD	3 (2.1%)	3 (2.3%)	0 (0.0%)	1.000
Liver cirrhosis	4 (2.8%)	4 (3.1%)	0 (0.0%)	1.000
Congestive heart failure	14 (9.9%)	14 (10.9%)	0 (0.0%)	0.609
Malignancy	5 (3.5%)	4 (3.1%)	1 (8.3%)	0.363
Laboratory data at admission				
Pre-operative serum creatinine (mg/dL)	1.2 ± 0.3	1.2 ± 0.3	1.1 ± 0.4	0.494
eGFR (MDRD) (ml/min/1.73 m^2^)	64.1 ± 20.6	64.1 ± 20.4	64.6 ± 23.4	0.934
Hemoglobin (g/dL)	13.0 ± 2.0	13.2 ± 2.0	11.6 ± 2.2	0.009
Albumin (g/dL)	4.2 ± 0.5	4.3 ± 0.5	4.0 ± 0.7	0.043
LVEF <55%	33 (23.4%)	30 (23.3%)	3 (25.0%)	0.999
Perioperative condition				
Inotropic equivalents	5.41 ± 5.76	4.86 ± 4.53	11.23 ± 11.92	0.092
Presence of CPB	87 (61.7%)	78 (60.5%)	9 (75.0%)	0.372
CPB time (min)	106.6 ± 98.9	103.2 ± 97.4	143.3 ± 112.0	0.200
Presence of Crossclamp	71 (50.4%)	63 (48.8%)	8 (66.7%)	0.379
Clamp time (min)	57.4 ± 65.1	55.7 ± 65.2	78.2 ± 63.8	0.295
Operative method				
CABG	72 (51.1%)	68 (52.7%)	4 (33.3%)	0.326
Valve	60 (42.6%)	53 (41.1%)	7 (58.3%)	0.395
Aorta	19 (13.5%)	17 (13.2%)	2 (16.7%)	0.665
length of admission (days)	21.3 ± 20.4	20.4 ± 19.9	30.8 ± 24.4	0.093
length of ICU admission (days)	3.8 ± 3.3	3.4 ± 2.8	7.3 ± 5.3	0.030
SOFA score	6.0 ± 2.8	5.8 ± 2.7	8.2 ± 3.6	0.008
Mortality	8 (5.7%)	3 (2.3%)	5 (41.7%)	<0.001

Continuous data are expressed as mean ± SD; nominal data are expressed as *n* (%).

BMI, body-mass index; COPD, chronic obstructive pulmonary disease; MDRD, Modification of Diet in Renal Disease; CPB, cardiopulmonary bypass; CABG, coronary artery bypass graft; SOFA, Sequential Organ Failure Assessment.

**Table 2:** Area Under the ROC curves for advanced (stage 2 or 3) AKI, or Composite Outcome.

	T3	T6	T9	T12	T24
Advanced AKI					
α-GST	0.477 (0.267–0.686)	0.489 (0.301–0.678)	0.508 (0.349–0.668)	0.395 (0.205–0.584)	0.604 (0.416–0.791)
adjusted α-GST^$^	0.395 (0.215–0.575)	0.435 (0.271–0.599)	0.448 (0.297–0.599)	0.366 (0.204–0.529)	0.515 (0.359–0.671)
π-GST	0.784 (0.673–0.895)^#^	0.649 (0.456–0.843)	0.767 (0.610–0.924)^#^	0.552 (0.360–0.743)	0.597 (0.414–0.780)
adjusted π-GST^$^	0.675 (0.546–0.804)*	0.529 (0.369–0.688)	0.587 (0.426–0.749)	0.384 (0.221–0.547)	0.413 (0.273–0.554)
Composite outcome					
α-GST	0.553 (0.366–0.739)	0.533 (0.372–0.693)	0.537 (0.398–0.676)	0.451 (0.286–0.616)	0.619 (0.462–0.775)
adjusted α-GST^$^	0.455 (0.291–0.620)	0.448 (0.298–0.597)	0.454 (0.314–0.593)	0.397 (0.251–0.543)	0.513 (0.379–0.647)
π-GST	0.783 (0.682–0.884)^#^	0.675 (0.512–0.837)*	0.763 (0.626–0.900)^#^	0.603 (0.432–0.774)	0.619 (0.461–0.777)
adjusted π-GST^$^	0.670 (0.549–0.792)*	0.521 (0.375–0.667)	0.565 (0.415–0.714)	0.407 (0.255–0.558)	0.413 (0.280–0.545)

Expressed as AUC (95% confidence interval); *p < 0.05, ^#^p < 0.005 against AUC = 0.5 by C statistics.

^$^concentration divided by urine creatinine level.

GST, glutathione S-transferase.

**labeled as 3 hours [T3], 6 hours [T6], 9 hours [T9], 12 hours later [T12], and 24 hours post-surgery [T24].

**Table 3:** Multivariable risk model for advanced AKI or composite outcome (advanced AKI or in-hospital death).

	For advanced AKI			For composite outcome		
Independent variables	Odds Ratio	95% CI	p	Odds Ratio	95% CI	p
π-GST*	1.610	1.046–2.551	0.033	1.618	1.135–2.366	0.009
Inotropic equivalents	1.182	1.020–1.257	0.030	1.109	1.018–1.228	0.029
Hemoglobin	0.657	0.431–0.949	0.034	—	—	—
BMI >32	18.205	1.392–220.821	0.020	—	—	—

AKI, acute kidney injury; BMI, body mass index, GST, glutathione S-transferase, CI, confidence interval.

*at 3 hours post surgery.
